# Correlation between polymorphisms of the NR3C1 gene and glucocorticoid effectiveness in patients with pemphigus vulgaris

**DOI:** 10.1038/s41598-017-12255-0

**Published:** 2017-09-19

**Authors:** Si-Yue Fang, Chun-Lei Li, Xiao-Song Liu, Feng Chen, Hong Hua

**Affiliations:** 1 Department of Oral Medicine, Peking University School and Hospital of Stomatology, Beijing, China; 2 Department of Stomatology, Dongzhimen Hospital Affiliated to Beijing University of Chinese Medicine, Beijing, China; 3 Central Laboratory, Peking University School and Hospital of Stomatology, Beijing, China

## Abstract

Glucocorticoid (GC) resistance is the major obscule in the treatment of pemphigus vulgaris (PV) for both patients and clinicans with unclear mechanism. A hypotheis for this resistance is the mutations or polymorphisms present in the nuclear receptor subfamily 3, group C, member 1 (NR3C1) gene that encodes receptors for steroid hormones. This study aimed to investigate the association between NR3C1 gene polymorphisms and GC effectiveness in PV patients. 94 PV patients (64 GC-sensitive and 30 GC-resistant) and 100 healthy volunteers were enrolled in this case-control study. The genotyping of single nucleotide polymorphisms (SNPs) in BCL1, Arg23Lys, Asn363Ser 1548 t-insert, and le747Met, together with tag-SNP sites of the NR3C1 gene were evaluated. No significant differences were observed in genotypic and allelic frequencies of the 16 SNPs between PV patients and healthy volunteers. However, SNPs rs 11745958 C/T (OR: 8.95) and rs17209237 A/G (OR: 4.07) may be associated with an increased risk of GC resistance, while rs 33388 A/T (OR: 0.45) and rs7701443 A/G (OR: 0.51) may indicate a decreased risk of GC resistance in PV patients. NR3C1 gene variation may be associated with GC resistance in PV patients. More extensive genetic analyses and mechanistic analysis are required for further exploration.

## Introduction

Pemphigus is a rare autoimmune disorder characterized by extensive blistering and erosions on the skin and mucosa caused by autoantibodies against desmoglein-1 (Dsg) and/or −3, which are major components of desmosomes; this damage leads to the histological observation of the detachment of epidermal cells^1^. Pemphigus vulgaris (PV) is one of the major forms of pemphigus, which affects the skin and mucosal membranes causing histologically acanthosis and blisters and/or erosions^2^. PV is also the most common form of pemphigus in the oral cavity. Moreover, the incidence of PV is estimated to be 0.6 to 6.8 new cases per million people per year^3^.

Pemphigus is a potentially fatal condition; however with the introduction of glucocorticoids (GC) in the 1950s, the mortality rate dramatically decreased from 75% to 30%^4^. Moreover, the more recently reported mortality rate was only 12%, and was primarily attributed to complications arising from the long-term use of immunosuppressive agents^5^. The therapeutic strategy of PV is focused on blocking the production of autoantibodies and certain cytokines such as BAFF (a member of the TNF superfamily), TNF-α, CD20,CD154, etc^6^. GC is recommended as the first-line remedy for pemphigus^6,7^ due to the acknowledged benefits of GCs in inducing clinical remission of most PV patients; however, a small but significant number of patients exhibit a poor or complete lack of response to GC, known as “GC resistance”^8,9^.

In general, the effect of GCs is mediated by its binding to the glucocorticoid receptor (GR)^10^. Thus, an alteration in the GR might contribute to the observed variation in the responses to GC. GRs are encoded by the nuclear receptor subfamily 3, group C, member 1 (NR3C1) gene, which resides on chromosome 5q31–32, contains 10 exons, and codes for 777 amino acids^11^. Indeed, several polymorphisms and mutations in NR3C1 have also been investigated in other disorders, including digestive tract, renal, and cardiovascular diseases^12–14^.

GC resistance or insensitivity is a major obstacle for the treatment of PV. However, the molecular mechanisms involved in GC resistance to PV have yet to be comprehensively investigated. This study aimed to evaluate the correlation between SNPs of the NR3C1 gene and GC resistance in PV patients.

## Results

### Characteristics of the enrolled subjects

A total of 94 patients with a confirmed diagnosis of PV (28.72% male and 71.28% female; mean age: 51.76 ± 12.33 y) and 100 healthy subjects (29% male and 71% female; mean age: 42.19 ± 10.12 y) were investigated in our study. Among the PV patients, 64 were GC-sensitive (28.13% male and 71.87% female; mean age: 51.53 ± 12.79 y) and 30 were GC-resistant (30% male and 70% female; mean age 52.23 ± 11.48 y). The subjects were appropriately age and sex matched.

### Allele and genotyping analysis

Among the 16 SNP sites, BCL1, Arg23Lys, 1548 t-insert, Asn363Ser, and Ile747Met failed to detect an SNP. The remaining 11 SNP sites in the control group were in HWE (*P* > 0.05).

No statistically significant differences were found regarding the genotype frequency and alleles in the 11 SNP sites between the PV patients and healthy control subjects (Table 1). Four SNP sites (rs11745958 C/T, rs33388 A/T, rs7701443 A/G, and rs17209237 A/G) exhibited significant differences between the GC sensitive and GC resistant groups (Table 2).

**Table 1:** Allele and genotype distributions in PV patients and controls.

	Control (%)	PV (%)	PV vs. Control P value	OR (95% CI)
**rs11745958**				
Allele				
C	174 (90.63%)	88 (84.62%)	0.122	0.57 (0.28–1.17)
T	18 (9.37%)	16 (15.38%)		
Genotype				
CC	78 (81.25%)	36 (69.23%)	0.097	
CT	18 (18.75)	16 (30.77%)		
**rs17209237**				
Allele				
A	153 (85.00%)	78 (76.47%)	0.074	0.57 (0.31–1.06)
G	27 (15.00%)	24 (23.53%)		
Genotype				
AA	65 (72.22%)	27 (52.94%)	0.02	
GG	2 (0.02%)	0 (0)		
AG	23 (25.56%)	24 (47.06%)		
**rs4607376**				
Allele				
A	86 (45.26%)	43 (41.35%)	0.518	0.85 (0.52–1.38)
G	104 (54.74%)	61 (58.65%)		
Genotype				
AA	19 (20.00%)	9 (17.31%)	0.798	
GG	28 (29.47%)	18 (34.62%)		
AG	48 (50.53%)	25 (48.08%)		
**rs7701443**				
Allele				
A	66 (34.02%)	33 (31.73%)	0.689	0.90 (0.54–1.50)
G	128 (65.98%)	71 (68.27%)		
Genotype				
AA	8 (8.25%)	5 (9.62%)	0.696	
GG	39 (40.21%)	24 (46.15%)		
AG	50 (51.55%)	23 (44.23%)		
**rs6865292**				
Allele				
C	55 (28.35%)	28 (26.92%)	0.793	0.93 (0.54–1.59)
T	139 (71.65%)	76 (73.08%)		
Genotype				
CC	8 (8.25%)	5 (9.62%)	0.793	
TT	50 (51.55%)	29 (55.77%)		
CT	39 (40.21%)	18 (34.61%)		
**rs117100234**				
Allele				
A	34 (17.89%)	19 (18.27%)	0.936	1.02 (0.55–1.91)
C	156 (82.11%)	85 (81.73%)		
Genotype				
AA	5 (5.26%)	1 (0.01%)	0.435	
CC	66 (69.47%)	34 (65.38%)		
AC	24 (25.27%)	17 (32.69%)		
**rs4912905**				
Allele				
C	73 (40.56%)	45 (44.12%)	0.560	1.16 (0.71–1.89)
G	107 (59.44%)	57 (55.88%)		
Genotype				
CC	15 (16.67%)	7 (13.73%)	0.322	
GG	32 (35.56%)	13 (25.49%)		
CG	43 (47.78%)	31 (60.78%)		
**rs4912905**				
Allele				
A	101 (54.89%)	62 (59.62%)	0.437	1.21 (0.74–1.97)
G	83 (45.11%)	42 (40.38%)		
Genotype				
AA	28 (30.43%)	17 (32.69%)	0.558	
GG	19 (20.65%)	7 (13.46%)		
AG	45 (48.92%)	28 (53.85%)		
**rs2963155**				
Allele				
A	157 (80.93%)	86 (84.31%)	0.470	1.27 (0.67–2.41)
G	37 (19.07%)	16 (15.69%)		
Genotype				
AA	61 (62.89%)	36 (70.59%)	0.530	
GG	1 (1.03%)	1 (1.96%)		
AG	35 (36.08%)	14 (27.45%)		
**rs33388**				
Allele				
A	31 (16.67%)	22 (21.15%)	0.343	1.34 (0.73–2.46)
T	155 (83.33%)	82 (78.85%)		
Genotype				
AA	2 (2.15%)	0 (0)	0.174	
TT	64 (68.82%)	30 (57.69%)		
AT	27 (29.03%)	22 (42.31%)		
**rs7719514**				
Allele				
A	24 (13.48%)	21 (20.59%)	0.12	1.66 (0.87–3.17)
G	154 (86.52%)	81 (79.41%)		
Genotype				
AA	0 (0)	1 (1.96%)	0.168	
GG	65 (73.03%)	31 (60.78%)		
AG	24 (24.97%)	19 (37.25%)

**Table 2:** Allele and genotype distributions in GC-sensitive and GC-resistant patients.

Polymorphisms	GC-resistant (%)	GC-sensitive (%)	P value	OR (95% CI)
**rs33388**				
Allele				
A	19 (31.67%)	22 (17.19%)	**0**.**025**	0.45 (0.22–0.91)
T	41 (68.33%)	106 (82.81%)		
Genotype				
AA	0 (0)	3 (4.69%)	**0**.**001**	
TT	11 (33.67%)	45 (70.31%)		
AT	19 (63.33%)	16 (25.00%)		
**rs7701443**				
Allele				
A	26 (43.33%)	36 (28.13%)	**0**.**039**	0.51 (0.27–0.97)
G	34 (56.67%)	92 (71.87%)		
Genotype				
AA	3 (10.00%)	5 (7.81%)	**0**.**033**	
GG	7 (23.33%)	33 (51.56%)		
AG	20 (66.67%)	26 (40.63%)		
**rs4607376**				
Allele				
A	29 (48.33%)	42 (32.81%)	0.041	0.52 (0.28–0.98)
G	31 (51.67%)	86 (67.19%)		
Genotype				
AA	6 (20.00%)	7 (10.94%)	0.105	
GG	7 (23.33%)	29 (45.31%)		
AG	17 (56.67%)	28 (43.75%)		
**rs17100234**				
Allele				
A	8 (13.33%)	15 (11.72%)	0.074	0.57 (0.31–1.06)
C	52 (86.67%)	113 (88.28%)		
Genotype				
AA	1 (3.33%)	2 (3.13%)	0.944	
CC	23 (76.67%)	51 (79.69%)		
AC	6 (20.00%)	11 (17.18%)		
**rs4912911**				
Allele				
A	38 (63.33%)	71 (55.47%)	0.308	0.72 (0.38–1.35)
G	22 (36.67%)	57 (44.53%)		
Genotype				
AA	11 (36.67%)	20 (31.25%)	0.459	
GG	3 (10.00%)	13 (20.31%)		
AG	16 (53.33%)	31 (48.44%)		
**rs2963155**				
Allele				
A	47 (78.33%)	105 (83.33%)	0.518	0.85 (0.52–1.38)
G	13 (21.67%)	21 (16.67%)		
Genotype				
AA	17 (56.67%)	45 (71.43%)	0.097	
GG	0 (0)	3 (4.76%)		
AG	13 (43.33%)	15 (23.81%)		
**rs7719514**				
Allele				
A	11 (18.97%)	27 (21.09%)	0.739	1.14 (0.52–2.50)
G	47 (81.03%)	101 (78.91%)		
Genotype				
AA	0 (0)	3 (4.69%)	0.470	
GG	18 (62.07%)	40 (62.50%)		
AG	11 (37.93%)	21 (32.81%)		
**rs6865292**				
Allele				
C	14 (23.33%)	35 (27.34%)	0.559	1.24 (0.60–2.52)
T	46 (76.67%)	93 (72.66%)		
Genotype				
CC	1 (3.33%)	7 (10.94%)	0.430	
TT	17 (56.67%)	36 (56.25%)		
CT	12 (40.00%)	21 (32.81%)		
**rs4912905**				
Allele				
C	20 (34.48%)	53 (42.06%)	0.329	1.38 (0.72–2.63)
G	38 (65.52%)	73 (57.94%)		
Genotype				
CC	2 (6.90%)	10 (15.87%)	0.477	
GG	11 (37.93%)	20 (31.75%)		
CG	16 (55.17%)	33 (52.38%)		
**rs17209237**				
Allele				
A	42 (70.00%)	114 (90.48%)	**<0**.**001**	4.07 (1.81–9.17)
G	18 (30.00%)	12 (9.52%)		
Genotype				
AA	12 (40.00%)	52 (82.54%)	**<0**.**001**	
GG	0 (0)	1 (1.59%)		
AG	18 (60.00%)	10 (15.84%)		
**rs11745958**				
Allele				
C	44 (73.33%)	123 (96.09%)	**<0**.**001**	8.95 (3.09–25.86)
T	16 (26.67%)	5 (3.91%)		
Genotype				
CC	14 (46.67%)	59 (92.19%)	**<0**.**001**	
CT	16 (53.33%)	5 (7.81%)		

For rs11745958, the frequency of the C allele (73.33%, *P* < 0.001) and CC genotype (46.67%, *P* < 0.001) in the GC resistant group was lower than that in the GC sensitive group (96.09% and 92.19%, respectively), which was associated with the increased risk of GC resistance in the subjects (OR: 8.95; CI: 3.09–25.86).

For rs33388, the frequency of the A allele (31.67%, *P* < 0.05) and AT genotype (63.33%, *P* = 0.001) in the GC resistant group was higher than that in GC sensitive group (17.19% and 25.00%, respectively), which was associated with a lower risk of GC resistance in the subjects (OR: 0.4; 95% CI: 0.22–0.91).

For rs7701443, the frequency of the A allele (43.33%, *P* < 0.05) and AA and AG genotype (10.00% and 66.67%, *P* < 0.05) in the GC resistant group was higher than that in the GC sensitive group (28.13%, 7.81%, and 40.63%, respectively), which was associated with lower risk of GC resistance in the subjects (OR: 0.51; 95% CI: 0.27–0.97).

For rs17209237, the frequency of the A allele (70.00%, *P* < 0.001) and AA genotype (40.00%, *P* < 0.001) in the GC resistant group was lower than that in the GC sensitive group (90.84% and 82.54%, respectively), which was associated with an increased risk of GC resistance in the subjects (OR: 4.07; 95% CI: 1.81–9.17).

### Linkage disequilibrium analysis

**Figure 1:**
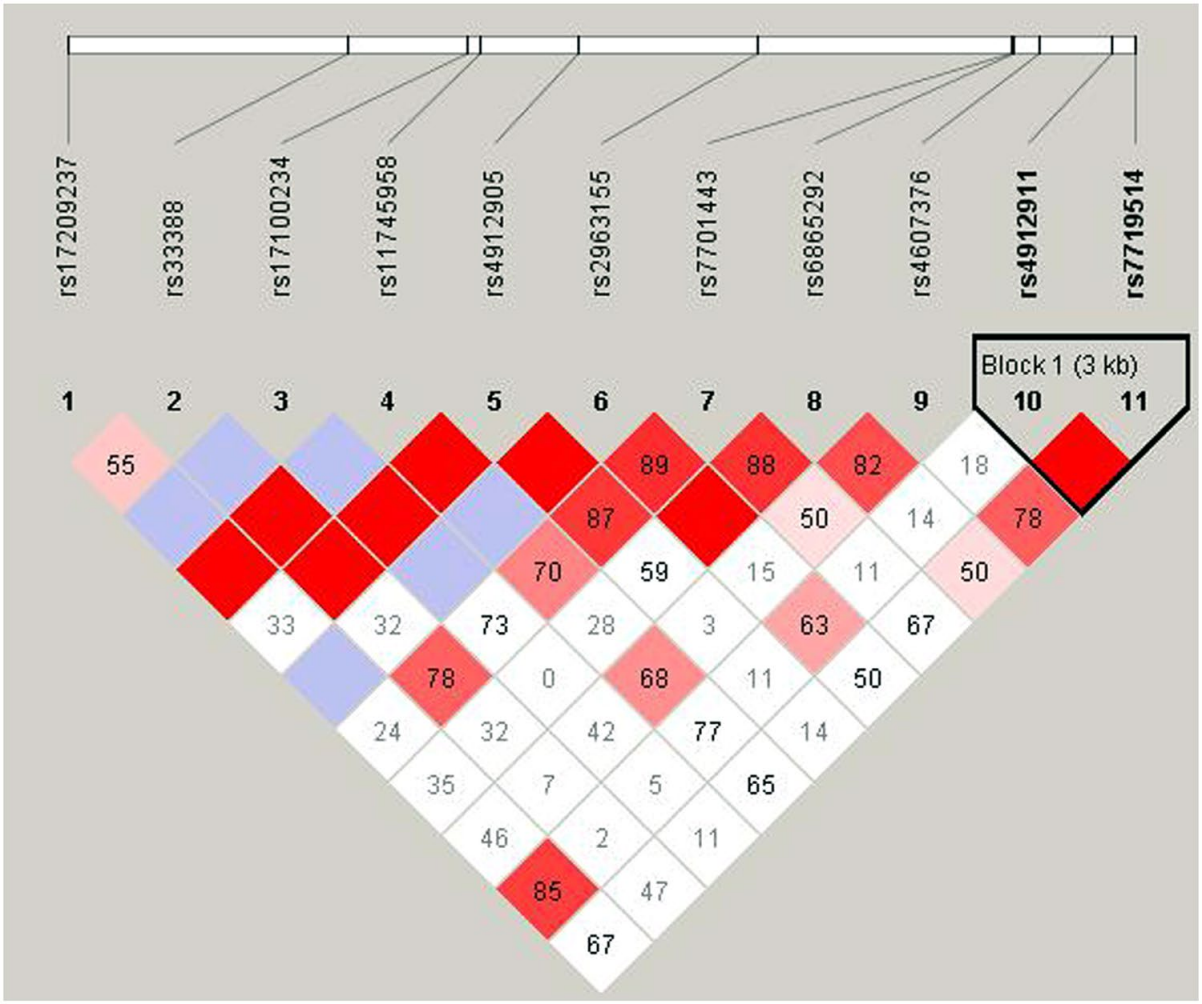
The pattern of linkage disequilibrium (LD) in the single nucleotide polymorphism sites. LD between the two variants are denoted by color: blue/gray = no LD; white = limited color; light red to dark red = medium to strong LD, respectively. The values for LD are presented within each square.

The linkage disequilibrium was analyzed among the 11 SNP sites using haploview software, and a haploblock between rs4912911 and rs7719514 was identified (Fig. 1). A Pearson’s chi-square test was then used to detect the haplotype distribution of the haploblock between GC-sensitive and GC-resistant groups; however, no statistically significant difference was observed (Table 3).

**Table 3:** Haplotype distribution in the haploblock between GC-sensitive and GC-resistant groups.

Block	Freq.	GC-resistant, GC-sensitive Ratio Counts	GC-resistant, GC-sensitive Frequencies	Chi Square	P Value
AG	0.58	71.0: 57.0, 38.0: 22.0	0.555, 0.633	1.037	0.3085
GG	0.215	30.0: 98.0, 10.5: 49.5	0.234, 0.175	0.846	0.3578
GA	0.205	27.0: 101.0, 11.5: 48.5	0.211, 0.191	0.095	0.7576

## Discussion

Pemphigus is a severe chronic autoimmune disorder with an unknown pathogenesis, for which therapeutic management is centered around the use of immunosuppressive agents (e.g., GC, cyclophosphamide, azathioprine, and rituximab) to inhibit the production of autoantibodies and inflammatory cytokines^6,15^. The first-line treatment regimens consist of high-dose systemic GC. However, the limitation of this treatment is the development of GC resistance due to a partial or complete inability of GC to exert its effect on target tissues, which is a major concern for both patients and clinicians. In addition, there is limited evidence regarding GC resistance in PV had been introduced^16^. GR is an important candidate for GC resistance and polymorphisms in the GR gene, NR3C1, have also been investigated in the context of several disorders and healths^17,18^.

However, no information regarding a correlation between NR3C1 SNPs and GC effectiveness in PV patients has been reported to date.

In our study, 94 PV patients, including 64 GC-sensitive and 30 GC-resistant subjects and 100 healthy volunteers, were enrolled to detect the association of 16 SNPs of the NR3C1 gene and GC effectiveness. No differences regarding the allele frequencies and genotyping of these SNP sites were found between the PV patients and healthy volunteers.

Four SNP sites (rs11745958 C/T, rs33388 A/T, rs7701443 A/G, and rs17209237 A/G) exhibited significant differences between the GC sensitive and GC resistant groups. In particular, rs11745958 C/T and rs17209237 A/G may be related to a decreased response to GC therapy, while rs33388 A/T and rs7701443 A/G may be associated with a favorable response to GC therapy in PV patients. Furthermore, the effects of the four SNP sites were investigated using an information database. One of these SNP sites (rs33388), exhibited potential transcription factor binding sites (TFBS), to which various transcription factors may bind, including activatorprotein-1 (AP-1) and sterol regulatory element binding protein (SREBP), which are both related to GC sensitivity^19,20^. However, the role of these transcription factors in the effectiveness of GC in PV patients requires further exploration.

Previous studies have indicated the relationship between altered GC sensitivity mediated by BclI and ER22/23EK polymorphism of GR gene and changes in body composiiton and metabolic parameters in healthy subjects^17,18^. Unlike this findings, BclI SNPs showed no statistical difference between subjects in our study. It may attribute to the different subjects selection (healthy voluteers vs. PV patienst) and methodology.

The present study presents evidence of an association between NR3C1 SNPs and GC effectiveness in PV patients using a case-control study. Due to the limitations of a small sample size and SNP selection, these results only provided preliminary conclusions. The influence of these SNPs mutations (rs11745958 C/T, rs17209237 A/G, rs33388 A/T and rs7701443 A/G) on GC binding and downstream signaling pathway, the insilico analysis to predict receptor affinity for these SNPs are also needed to intensively investigate the relationship between NR3C1 gene and GC efficacy in our furture study.

To sum up, our finding suggested that the polymorphisms of the NR3C1 gene was associated to GC efficacy, further study involving a fine-mapping analysis of the NR3C1 genes and its mechanism are required to fully interpret the relationship between NR3C1 gene mutations and GC effectiveness.

## Materials and Methods

### Ethical approval

This study was approved by the Peking University Institutional Review Board (ref. No. PKUSSIRB-2013033) and all methods were performed in accordane with the relevant guidelines and regulations. Each subject agreed and signed the infromed consent prior to the study.

### Patients

A total of 94 PV patients referred to the Department of Oral Medicine, Peking University School and Hospital of Stomatology from Jan 2004 to Dec 2010 were enrolled in the case-control study. The subjects were recruited in accordance with the following inclusion and exclusion criteria. Inclusion criteria: 1) PV patients with a confirmed diagnosis via clinical and histological findings, as well as direct immunofluorescence (DIF) or an ELISA; 2) PV patients who had not previously received GC treatment at the time of the study; and 3) the treatment duration was longer than six months. Exclusion criteria: 1) PV patients who had previously received GC treatment at the time of the study; 2) patients with other systemic diseases (e.g., tuberculosis) who were not suitable for GC treatment; and (3) patients who received other immunosuppressant agents other than GC.

The diagnosis was made based on clinical manifestation and at least two laboratory tests, including exfoli-cytology examination, histological findings, indirect immunofluorescence or an ELISA^16^. Following the diagnosis, all patients recieved GC from 0.50–0.75 mg/kg/day as suggested by the guidelines^21^. Patients were classified as being either poor or good responders to GC therapy. A poor response was defined as the consistent persistence of oral or skin lesions or the appearance of new blisters in the oral cavity or skin despite prednisone or prednisolone treatment up to 0.75 mg/kg/day for four weeks^22^. We also enrolled 100 healthy individuals as controls.

### SNP selection

There were five SNP sites in the coding region of the NR3C1 gene (BCL1, Arg23Lys, Asn363Ser, 1548 t-insert, and le747Met) related to amino acid changes that were chosen in the present study. The chosen of SNPs was based on the published data which were reported to be associated with GC effectiveness in health and patients^12,14,17,18,23^.

In addition, 11 Tag-SNP sites, including rs11745958, rs17100234, rs17209237, rs2963155, rs33388, rs4607376, rs4912905, rs4912911, rs6865292, rs7701443, and rs7719514 were selected via a shared database (http://www.ncbi.nlm).

### Isolation of DNA and genotyping by Matrix-assisted laser desorption/ionization time-of-flight mass spectrometry (MALDI-TOF-MS)

Whole blood samples were collected and transferred to test tubes containing ethylenediamine tetra-acetic acid (EDTA) from all subjects. The genomic DNA was then isolated from the blood using a whole blood genetic DNA extraction kit (BioTeke, Beijing, China). An OD assay was used to calculate the concentration of the DNA products. The samples were subjected to MALDI-TOF MS for a genotype analysis of the SNP sites.

### Statistical analysis

All data were inputted in parallel into EpiData 3.0 software and EXCEL 2010, and analyzed using IBM SPSS statistical software (version 13.0, IBM Crop, Armonk:NY, USA). Differences in the demographic and clinical characteristics were evaluated using a chi-square test. The association between each SNP site and GC resistance phenotype was estimated by calculating the odds ratio (OD) and 95% confidence intervals (CIs) via logistic regression analyses. A Hardy-Weinbery equilibrium (HWE) test, haplotype frequency calculation, and linkage disequilibrium were performed using HaploView software. The significance level was set at *P* < 0.05.
